# Reactive Granulomatous Dermatitis: A Descriptive Study of 10 Patients

**DOI:** 10.1177/12034754231220937

**Published:** 2024-01-16

**Authors:** Maude Lagacé, Laurence Mainville, Marie-Claude Dionne

**Affiliations:** 1 Université Laval Faculté de Médecine, Québec, QC, Canada; 2 Laval University, Québec, QC, Canada

**Keywords:** reactive granulomatous dermatitis, interstitial granulomatous dermatitis, palisaded neutrophilic and granulomatous dermatitis, interstitial granulomatous drug reaction

## Abstract

**Background:**

Reactive granulomatous dermatitis (RGD) is a rare and misunderstood skin disorder. It includes interstitial granulomatous dermatitis and palisaded neutrophilic and granulomatous dermatitis: 2 entities of the same spectrum. Multiple associations are described with RGD in the literature, including autoimmune diseases, malignancy, and drugs.

**Objective:**

To report and describe the suspected associations with RGD at the time of diagnosis and in the following year.

**Methods:**

We retrieved and described cases of RGD confirmed by skin biopsy and clinicopathologic correlation. All patients were evaluated in the *Centre Hospitalier Universitaire de Québec*—*Université Laval* between January 2000 and December 2020. Collected data include the systemic diseases (autoimmune disease, malignancy) and suspected drugs, in addition to the clinical presentation and prescribed treatments.

**Results:**

Out of the 10 patients with RGD, 7 patients were known to have an autoimmune disease at the time of diagnosis. They either had inflammatory arthritis (3/10) or inflammatory bowel disease (4/10). There was a clinical suspicion of a possible association with a tumor necrosis factor (TNF) inhibitor in 2 of these 7 patients. Among the 3 patients with idiopathic RGD at the time of diagnosis, 1 patient developed a high-grade B-cell lymphoma 6 months later. There was no new association identified in the following year for patients with a known autoimmune condition.

**Conclusion:**

This descriptive study supports RGD and its previously described systemic associations, particularly autoimmune diseases, malignancy, and certain drugs (specifically TNF inhibitors). The majority of patients already had one of these associations identified at the time of histopathological diagnosis.

## Introduction

Reactive granulomatous dermatitis (RGD) is a rare disease whose pathogenesis has not yet been fully elucidated. This entity is part of a group of non-infectious granulomatous diseases including sarcoidosis, granuloma annulare, annular elastolytic giant cell granuloma, and necrobiosis lipoidica. RGD includes interstitial granulomatous dermatitis (IGD) and palisaded neutrophilic and granulomatous dermatitis (PNGD), in addition to interstitial granulomatous drug reaction (IGDR).^1,2^ Indeed, given the significant overlap in their clinical presentations, their histological findings, and their underlying conditions, they are considered a continuum by many authors. The growing recognition that RGD is an all-encompassing term reduced previous confusion in the scientific literature surrounding this nomenclature.

RGD is found in association with various systemic diseases, such as autoimmune diseases (eg, inflammatory arthritis, connective tissue disease, vasculitis),^1-3^ malignancy (especially hematologic neoplasms),^4^ certain drugs [eg, tumor necrosis factor (TNF) inhibitors, furosemide, angiotensin-converting enzyme inhibitors],^1,5^ and rare infections (coccidioidomycosis and borreliosis).^6,7^ The majority of patients had a systemic association prior to diagnosis.^2^ However, it is unclear which investigations should be carried out and if new associations were subsequently identified. Thus, the objective of this study is to describe cases of RGD with their possible underlying associations at the time of diagnosis and within the following year. It aims to support the associations previously described in the literature and possibly enlighten physicians on the relevant investigations to perform.

## Material and Methods

This study was approved by the Director of Professional Services of the Capitale-Nationale and the Ethics Committee to access patient files.

We describe confirmed cases of RGD seen between January 2000 and December 2020 in the *Centre Hospitalier Universitaire de Québec*—*Université Laval.* They were identified by the morphological codes used by the pathology department. Any histopathological diagnosis of granuloma other than RGD was excluded. All patients have confirmed clinicopathologic correlation of RGD by a qualified dermatologist who ultimately retained IGD, PNGD, and/or IGDR as the final diagnosis. Patients of all ages and genders were accepted.

The data were collected from electronic patient records. A retrospective chart review was conducted. Notably, histopathological diagnosis of RGD, demographic characteristics, clinical descriptions, identification of suspected association, and prescribed investigations were collected at the time of diagnosis. New systemic conditions identified within a 12-month period from the diagnosis were also retrieved.

## Results

Among the patients, 4/10 were diagnosed with IGD, 3/10 with PNGD, and 3/10 mentioned both. A majority of patients were female (8/10). Their ages ranged from 29 to 81, with a median age of 48 years. The clinical presentations and locations were highly variable between patients. One patient had a single large lesion, while others had multiple disseminated lesions. Lesions were mainly present on the trunk and limbs (8/10). Four patients had lesions on the hands. Only 1 patient had facial involvement. Table 1 summarizes the clinical characteristics of all 10 patients.

**Table 1.** Characteristics of 10 Patients With Reactive Granulomatous Dermatitis.

Patient	Diagnosis	Sex	Age	Clinical description	Affected sites
1	IGD	H	71	8 purple firm edematous papules	Back, right arm, and right hand
2	IGD	F	63	Multiple erythematous papules	Neck and anterior chest
3	IGD	F	81	Purpuric edematous nodules and plaques	Arms and legs
4	IGD-PNGD	H	66	Infiltrated erythematous plaques; linear or arciform	Back and right thigh
5	IDG-PNGD	F	35	Erythematous nodules	Elbows and proximal interphalangeal joints
6	IGD	F	47	Erythematous nodules	Fingers
7	PNGD	F	49	20 to 30 firm pink-purple papules and nodules	Arms and thighs
8	PNGD	F	29	Purple edematous papules and plaques with pseudo vesicles	Neck, anterior chest, upper limbs, and face
9	PNGD	F	29	Pink and purpuric edematous papules	Fingers
10	IGD-PNGD	F	45	Large cyanotic erythematous plaque	Left thigh and buttocks

Abbreviations: IGD, interstitial granulomatous dermatitis; PNGD, palisaded neutrophilic and granulomatous dermatitis.

At the time of diagnosis, 7/10 patients had a known autoimmune disease: 3 patients with seronegative arthritis and 4 patients with inflammatory bowel disease (3 patients had Crohn’s disease and 1 patient had ulcerative colitis). One patient with Crohn’s disease also had concomitant spondylitis. Furthermore, in 2 of the 7 patients with autoimmune conditions, there was a clinical suspicion of a possible association with a TNF inhibitor (infliximab and etanercept, respectively). In both cases, the clinicians could not determine whether the TNF inhibitor or the associated autoimmune disease was the precipitating factor. In one of these patients, the TNF inhibitor was changed to baricitinib, a Janus kinase inhibitor, with subsequent improvement of lesions. None of the 10 patients had a known malignancy at the time of RGD diagnosis.

Two out of 10 patients were not further investigated after skin biopsy. In 8/10 patients, conducted investigations varied and included complete blood count, anti-nuclear antibody, rheumatoid factor, extractable nuclear antigen, thyroid function tests, serum protein electrophoresis with immunofixation, sedimentation rate, and C-reactive protein. Within the 12-month period, only 1 patient had a new association possibly related to RGD. An 81-year-old woman was diagnosed with a high-grade B-cell lymphoma 6 months after the diagnosis of RGD and died shortly thereafter. This patient was 1 of the 3 patients categorized as idiopathic RGD at the time of diagnosis.

Prescribed treatments aimed to treat RGD and/or its clinically suspected underlying association. The majority of patients (8/10) were treated with topical corticosteroids. Response with this treatment ranged from resolution within 1 month for 1 patient, while 3 other patients still had recurrent plaques after 12 months from diagnosis. Concomitantly or subsequently, some patients received hydroxychloroquine, methotrexate, and oral corticosteroids with varying responses. The majority of patients still had lesions despite the use of these systemic treatments. Two patients started a new TNF inhibitor (adalimumab and infliximab), a drug paradoxically associated with RGD. In these 2 cases, no clinical improvement of RGD was observed during the 12 months following diagnosis.

As previously mentioned, a patient’s TNF inhibitor (etanercept), possibly thought to be a trigger, was changed to baricitinib. With this replacement and topical corticosteroids, resolution of RGD occurred within 3 months. The second patient with a clinical suspicion of TNF inhibitor-induced RGD continued to receive infliximab. He was treated only with topical corticosteroids, and the lesions persisted within 12 months of diagnosis.

Patients had variable evolutions. In 4/10, remission was observed in a few months, while 6/10 patients had no resolution during the 12-month period with recurrence of lesions. Table 2 summarizes the associations, investigations, and treatments for all 10 patients.

**Table 2.** Associated Diseases of 10 Patients With Reactive Granulomatous Dermatitis.

Patient	Association at the moment of diagnosis			Investigation after diagnosis	New association within 1 year of diagnosis	Treatments	Time between diagnosis and resolution of lesions
	Autoimmune diseases	Drug	Malignancy				
1	No	No	No	Yes	No	Topical corticosteroids	>12 months
2	Seronegative arthritis	TNF inhibitor (Etanercept)	No	No	No	Topical corticosteroids, baricitinib (for etanercept replacement)	3 months
3	No	No	No	Yes	High-grade B-cell lymphoma	No treatment	1 month
4	No	No	No	Yes	No	Topical corticosteroids	1 month
5	Seronegative arthritis	No	No	Yes	No	Topical corticosteroids, hydroxychloroquine	>12 months
6	Crohn’s-associated spondylitis	No	No	Yes	No	Topical corticosteroids, methotrexate, adalimumab	>12 months
7	Seronegative arthritis	No	No	No	No	Colchicine, hydroxychloroquine, methotrexate, oral corticosteroids	7 months
8	Crohn’s disease	No	No	Yes	No	Topical corticosteroids, hydroxychloroquine, dapsone, infliximab	>12 months
9	Ulcerative colitis	TNF inhibitor (Infliximab)	No	Yes	No	Topical corticosteroids	>12 months
10	Crohn’s disease	No	No	Yes	No	Topical and oral corticosteroids	>12 months

Abbreviation: TNF, tumor necrosis factor.

## Discussion

We describe 10 cases of RGD and their suspected systemic associations. The majority of patients (70% of cases) had an autoimmune disease upon diagnosis, either inflammatory arthritis or inflammatory bowel disease. Our results mirror the cases previously described in the literature. Indeed, in the recent study by Kumar et al, 76.9% of patients had an associated systemic disease before the development of RGD. In this same study, the most common association was a rheumatologic condition (52.3% of patients).^2^ Besides arthritis and inflammatory bowel disease, systemic lupus erythematosus, systemic sclerosis, ANCA vasculitis, Sjogren’s syndrome, and mixed cryoglobulinemia have also been described with RGD.^1-3,8-13^

Analogous to previous publications, TNF inhibitors could be a trigger for RGD.^14^ Out of the 10 patients in our study, 2 cases were possibly related to this medication. However, doubts remain about whether it is related to the active disease or the prescribed medication. Other drugs often associated with RGD are calcium channel blockers, angiotensin-converting enzyme inhibitors, statins, furosemide, and beta-blockers.^1,2,5^

No patient had malignancy at the time of diagnosis, but 1 patient was diagnosed with a high-grade B-cell lymphoma in the following months. The association with hematological neoplasms has also been noted in the literature, such as Hodgkin lymphomas, myelodysplastic syndromes, and acute myelocytic leukemia.^1,2,4,15^

In most cases, skin involvement did not resolve within the year following the diagnosis of RGD. These findings contrast with previous studies where PNGD patients had rapid resolution and IGD patients had spontaneous remission. For instance, Aloi et al^16^ reported 3 cases of RGD that resolved spontaneously over a period of 6 to 12 months. Several treatments are used to treat RGD and/or the underlying disease, the most common being topical corticosteroids.^2,17^ Therapeutic alternatives described in the literature include systemic corticosteroids, hydroxychloroquine, TNF inhibitors, colchicine, methotrexate, azathioprine, dapsone, cyclosporine, doxycycline, and intralesional corticosteroids.^2,3,18^ Baricitinib could be an additional therapeutic option.

Given the design of our study and our small population, we cannot recommend precise investigations in patients with RGD. Nevertheless, reported cases of RGD in patients with autoimmune diseases and hematological neoplasia should be kept in mind when considering further investigations, especially in cases where an underlying systemic disorder was not initially present at the time of diagnosis.

In conclusion, this descriptive study reflects the associations previously described with RGD, particularly autoimmune diseases, malignancy, and certain drugs (specifically inhibitors of TNF).^1,2^ Most patients already had an underlying systemic disease possibly associated with RGD at the time of histopathological diagnosis. Among the 3 patients without an initial identification of association, 2 patients remained idiopathic despite further investigations and 1 patient developed a lymphoma in the following months. An interesting avenue for future studies would be to do a longer follow-up of idiopathic cases to better characterize new associations, with particular attention to malignancy.

## References

[1] Rosenbach M English JC 3rd . Reactive granulomatous dermatitis: a review of palisaded neutrophilic and granulomatous dermatitis, interstitial granulomatous dermatitis, interstitial granulomatous drug reaction, and a proposed reclassification. Dermatol Clin. 2015;33:373-387. 26143420 10.1016/j.det.2015.03.005

[2] Bangalore Kumar A Lehman JS Johnson EF , et al. Reactive granulomatous dermatitis as a clinically relevant and unifying term: a retrospective review of clinical features, associated systemic diseases, histopathology and treatment for a series of 65 patients at Mayo Clinic. J Eur Acad Dermatol Venereol. 2022;36(12):2443-2450. 35535506 10.1111/jdv.18203 PMC9646920

[3] Rodríguez-Garijo N Bielsa I Mascaró JM Jr , et al. Reactive granulomatous dermatitis as a histological pattern including manifestations of interstitial granulomatous dermatitis and palisaded neutrophilic and granulomatous dermatitis: a study of 52 patients. J Eur Acad Dermatol Venereol. 2021;35(4):988-994. 33098595 10.1111/jdv.17010

[4] Pei S Hinshaw MA . Palisaded neutrophilic granulomatous dermatitis leading to diagnosis of Hodgkin lymphoma: report of rare case and literature review of paraneoplastic granulomatous dermatitides. Am J Dermatopathol. 2019;41(11):835-845. 30921008 10.1097/DAD.0000000000001411

[5] Magro CM Crowson AN Schapiro BL . The interstitial granulomatous drug reaction: a distinctive clinical and pathological entity. J Cutan Pathol. 1998;25:72-78. 9521495 10.1111/j.1600-0560.1998.tb01693.x

[6] Mangold AR DiCaudo DJ Blair JE Sekulic A . Chronic interstitial granulomatous dermatitis in coccidioidomycosis. Br J Dermatol. 2016;174(4):881-884. 26574343 10.1111/bjd.14295

[7] Badin DJ O’Hern K Simmons BJ Mann JA Momtahen S . Localized reactive granulomatous dermatitis secondary to erythema migrans. JAAD Case Rep. 2020;6(12):1236-1238. 33294552 10.1016/j.jdcr.2020.10.005 PMC7701020

[8] Stiff KM Cohen PR . Palisaded granulomatous dermatitis associated with ulcerative colitis: a comprehensive literature review. Cureus. 2017;9(1):e958. 10.7759/cureus.958 PMC5293147 28168136

[9] Jandali B Uthman I Abbas O . Interstitial granulomatous dermatitis associated with systemic lupus erythematosus: case report and review of the literature. Lupus. 2016;25(2):209-213. 26385222 10.1177/0961203315604908

[10] Jung KH Jeong S Kwon SR , et al. Palisaded neutrophilic granulomatous dermatitis in a patient with systemic sclerosis-rheumatoid arthritis overlap syndrome. Ann Dermatol. 2017;29(6):804-806. 29200777 10.5021/ad.2017.29.6.804 PMC5705370

[11] Akagawa M Hattori Y Mizutani Y Shu E Miyazaki T Seishima M . Palisaded neutrophilic and granulomatous dermatitis in a patient with granulomatosis with polyangiitis. Case Rep Dermatol. 2020;12(1):52-56. 32308576 10.1159/000506670 PMC7154257

[12] Sugioka K Goto H Sugita K Habe K Yamanaka K Yamamoto O . Palisaded neutrophilic granulomatous dermatitis, interstitial granulomatous dermatitis and IgA vasculitis associated with incomplete Sjögren’s syndrome. J Dermatol. 2021;48(4):556-558. 33406284 10.1111/1346-8138.15738

[13] Rato M Gil F Monteiro AF Aranha J Tavares E . Interstitial granulomatous dermatitis in a patient with chronic hepatitis C and mixed cryoglobulinemia. Dermatol Online J 2018;24:14. 29469770

[14] Deng A Harvey V Sina B , et al. Interstitial granulomatous dermatitis associated with the use of tumor necrosis factor alpha inhibitors. Arch Dermatol. 2006;142:198-202. 16490847 10.1001/archderm.142.2.198

[15] Weed J Ko C Stahl M , et al. Reactive granulomatous dermatitis presenting as subcutaneous nodules and cords in a patient with advanced myelodysplastic syndrome. Ann Hematol. 2017;96(6):1037-1039. 28220192 10.1007/s00277-017-2954-5

[16] Aloi F Tomasini C Pippione M . Interstitial granulomatous dermatitis with plaques. Am J Dermatopathol. 1999;21:320-323. 10446771 10.1097/00000372-199908000-00002

[17] Peroni A Colato C Schena D Gisondi P Girolomoni G . Interstitial granulomatous dermatitis: a distinct entity with characteristic histological and clinical pattern. Br J Dermatol. 2012;166:775-783. 22059717 10.1111/j.1365-2133.2011.10727.x

[18] Yang C Tang S Li S , et al. Underlying systemic diseases in interstitial granulomatous dermatitis and palisaded neutrophilic granulomatous dermatitis: a systematic review. Dermatology 2023;239(2):287-298. 36476409 10.1159/000527461

